# Antibiotic Resistance of *E. coli* Isolated From a Constructed Wetland Dominated by a Crow Roost, With Emphasis on ESBL and AmpC Containing *E. coli*

**DOI:** 10.3389/fmicb.2019.01034

**Published:** 2019-05-15

**Authors:** Keya Sen, Tanner Berglund, Marilia A. Soares, Babak Taheri, Yizheng Ma, Laura Khalil, Megan Fridge, Jingrang Lu, Robert J. Turner

**Affiliations:** ^1^Division of Biological Sciences, STEM, University of Washington, Bothell, WA, United States; ^2^Office of Research and Development, United States Environmental Protection Agency, Cincinnati, OH, United States; ^3^School of Interdisciplinary Arts and Sciences, University of Washington, Bothell, WA, United States

**Keywords:** wetland, crows, ST131, ESBL, multi-drug resistant *E. coli*, antibiotic resistant genes, *bla*_ctx-M_, *bla*_cmy-2_

## Abstract

Information on the dissemination of antibiotic resistance mechanisms in the environment as well as wild life is needed in North America. A constructed wetland (where ∼15,000 American crows roost) was sampled on the University of Washington Bothell Campus for the presence of antibiotic resistant *E. coli* (ARE). Crow droppings from individual birds and grab samples of water were collected in 2014–2015. *E. coli* were isolated by selective agar plating. The most frequent antibiotic resistance (AR) of the fecal isolates was to ampicillin (AMP) (53%), followed by amoxicillin-clavulanic acid (AMC) (45%), streptomycin (S) (40%), and nalidixic acid (NA) (33%). Water isolates had similar AR pattern and ∼40% were multidrug resistant. Isolates from water samples collected during storm events showed higher resistance than isolates from no rain days to tetracycline, AMP, AMC, NA, and gentamycin. Extended spectrum beta lactamase (ESBL) containing *E. coli* with the *bla*_ctx-M_ was found in three water and nine fecal isolates while *bla*_cmy-2_ in 19 water and 16 fecal isolates. Multilocus Sequence Typing analysis (MLST) yielded 13 and 12 different sequence types (STs) amongst fecal and water isolates, many of which could be correlated to livestock, bird, and humans. MLST identified ESBL *E. coli* belonging to the clinically relevant ST131 clone in six fecal and one water isolate. Three STs found in feces could be found in water on the same dates of collection but not subsequently. Thus, the strains do not appear to survive for long in the wetland. Phylogenetic analysis revealed similar distribution of the water and fecal isolates among the different phylo-groups, with the majority belonging to the commensal B1 phylo-group, followed by the pathogenic B2 phylo-group. This study demonstrates that corvids can be reservoirs and vectors of ARE and pathogenic *E. coli*, posing a significant environmental threat.

## Introduction

The spread of antimicrobial resistance has reached proportions of global magnitude and poses a threat to the effective treatment of several infectious diseases (Centers for Disease Control and Prevention [CDC], 2013; WHO, 2014). The environment is increasingly being recognized as a reservoir of antibiotic resistant (AR) bacteria as well as antibiotic resistant genes (ARG). Such resistance may arise by the release of fecal bacteria from humans and animals including birds, which then allows antibiotic resistance genes to be transferred to non-resistant indigenous microorganisms in the environment (Aminov, 2011; Guenther et al., 2011). Antibiotics or other chemicals and contaminants present in environmental matrices, contribute to this further by offering selective pressure, thus allowing for their survival and expansion (Martinez, 2009). Fecal contamination of surface water, river water, wetlands, and even drinking water have been implicated in the spread of such resistance (Baquero et al., 2008; Coleman et al., 2013; Li et al., 2014; Rodriguez-Mozaz et al., 2015; Vivant et al., 2016). On the other hand, constructed wetlands have also been shown to remove such bacteria (Ibekwe et al., 2016; Vivant et al., 2016).

Free living birds can be a significant contributor to the pollution of water bodies. Although they may not be directly exposed to antibiotics like humans or farm animals, they can acquire antibiotic resistance by being in close contact to humans, their farm animals and pets, and subsequently be vectors for their spread (Verbeek and Caffrey, 2002; Guenther et al., 2011; Jamborova et al., 2015). In addition, crows can acquire AR bacteria by foraging on a variety of wastes such as garbage dumps, hospital and animal wastes, and animal feed lots (Verbeek and Caffrey, 2002; Guenther et al., 2011). Several recent studies have reported crows and rooks shedding bacteria that were resistant to one or more antibiotics (Literak et al., 2007; Hasan et al., 2015; Jamborova et al., 2015, 2018). *E. coli*, which lives as a harmless commensal in the gut of all animal and birds, has proved to be not only an indicator of fecal coliform but also of antibiotic resistance present in the environment (van Den Bogaard et al., 2000; Dolejská et al., 2009; Guenther et al., 2011; Jamborova et al., 2015, 2018). From the United States, only one study investigating antibiotic resistance in *E. coli* in crows has been reported (Jamborova et al., 2017). In this study, which was a survey from four different states, 13% (*n* = 590) of *E. coli* from American crows (*Corvus brachyrhyncos*) possessed AmpC and ESBL phenotypes, while 15% (*n* = 590) were resistant to Ciprofloxacin (Jamborova et al., 2017). Two other studies reported on vancomycin resistant enterococci shed by crows in United States (Oravcova et al., 2014; Roberts et al., 2016). These studies specifically selected for cefotaxime or ciprofloxacin or vancomycin resistant bacteria. The overall antibiotic resistance pattern of the crow isolates was not reported.

In this study, samples collected within the University of Washington Bothell/Cascadia College (UWB/CC) campus (where more than 15,000 crows roost in the autumn and winter months) were tested for the resistance of *E. coli* isolates to thirteen antibiotics represented three classes of antibiotics. Extended Spectrum beta lactamase (ESBL) and AmpC beta lactamase containing *E. coli* were additionally targeted because the presence of these genes continue to hinder the efficacy of beta lactams (Pitout et al., 2007). The spread of ESBL resistance by crows has been documented in other parts of the United States, but not in Washington State (Jamborova et al., 2017). Multi Locus Sequence Typing (MLST) and phylogenetic characterization of the isolates was performed in order to have an idea of the source and pathogenicity of the isolates.

## Materials and Methods

### Sample Collections

**FIGURE 1:**
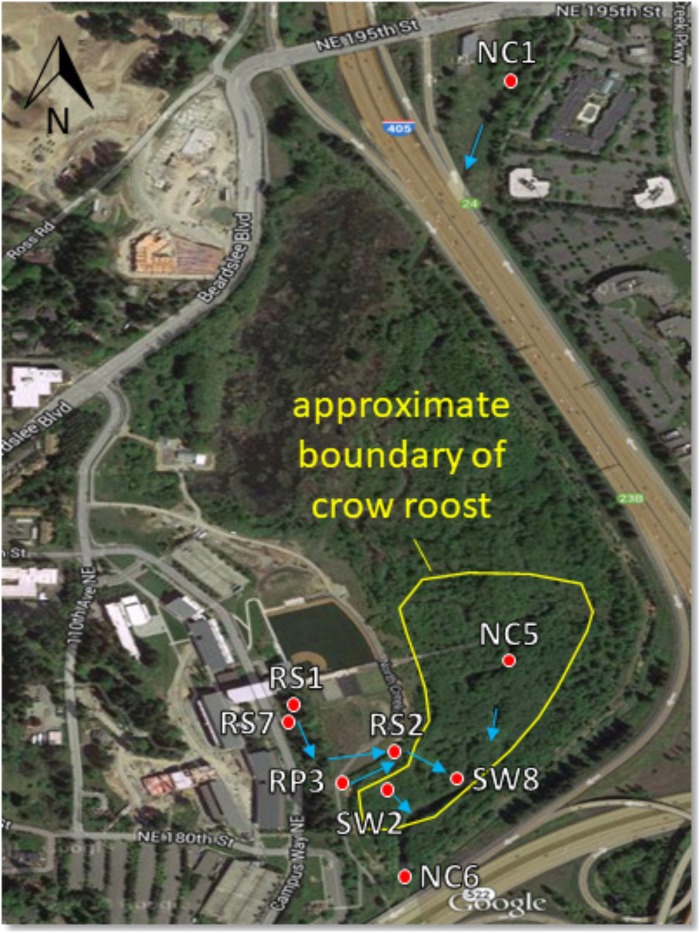
Sampling site map showing North Creek, the UW Bothell/Cascadia College campus, and the 58 acre restored floodplain wetland. Red dots indicate locations of surface water sampling sites. Blue arrows indicate direction of water flow. Water sampled at RS1, RS7, and RP3 flows to these locations in a series of catch basins and pipes from the upland (western) portion of the campus. The crow roost boundary fluctuates year to year, though the southern part by the sampling sites is relatively stable. Aerial photograph from Google.

All samples were collected within the 58-acre wetland restoration area of the UWB/CC campus. Begun in 1997 with the construction of campus, this restoration project converted pastureland and a straightened and deepened reach of North Creek into a more natural, meandering stream channel and a fully functioning forested floodplain ecosystem. It serves as a natural filter for campus stormwater runoff that is discharged in various locations to the wetland prior to flowing into North Creek (see Figure 1). The campus runoff contributes to the wetness of the wetland, as do a high water table, plentiful rain between October and June, and occasional flood events when North Creek spills over its banks (∼2–4 times a year).

Crow fecal samples were collected between August 2014 and April 2015 from the crow roost areas within the wetland. Samples were collected by spreading plastic sheets on the ground underneath the trees where the crows roosted in the evening. Fresh fecal samples from free flying crows were collected the following morning with sterile swabs and placed in sterile vials kept on ice as described previously (Sen et al., 2018). Sixty one samples were collected in five rounds of sampling. On the days that fecal samples were collected, surface water samples were also collected within the wetland at four different sites. Two sites, NC5 and SW2 were within the roost area while RP3 and NC6 were located in areas bordering the roost (Figure 1). Twenty water samples were collected altogether during this period. Water samples were collected again from June, 2016–April, 2017 from the sites designated as SW8, SW2, NC6, RS1, and RS2 to compare *E. coli* collected during “no rain” versus “rainy” days. The NC prefix of sampling sites indicates North Creek water. SW indicates a surface water tributary to North Creek. RS indicates discharge of campus runoff into a runoff bioswale. To qualify as a rainy day, more than 0.05 inches of cumulative rain for that day had to be recorded at the 21 Acres weather station approximately 1.5 miles away^1^. No rain days not only had no rain that day, but were also preceded by 72 h without rain.

#### Isolation and Enumeration of *E. coli*

Approximately 100 mg of fecal sample was diluted in 500 ml Phosphate Buffered Saline until a fluid suspension was obtained. Ten to twenty microliters were directly plated onto Eosin Methylene Blue (EMB) Agar and incubated at 37°C for 24 h. Colonies with metallic green sheen were isolated as putative *E. coli.* They were further verified by the presence of the malate dehydrogenase (*mdh)* gene as described below. From the 61 samples, 49 samples were positive for *E. coli*. Four isolates from each sample were stored at -70°C in Tryptic Soy Broth containing 16–20% glycerol until ready for use.

Water samples were collected in 120 ml IDEXX polyethylene terephthalate vessels and subsequently filtered through 0.45 micron Millipore S-Pak filters. *E. coli* and other coliform bacteria colonies were allowed to grow on the filters by placing them on m-ColiBlue24 broth following US EPA method 10029 (Hach Company 2018)^2^. Triplicate samples were collected at each site. Most of the water samples required dilution in order to generate countable filters.

Blue Colonies were counted for determination of total number of *E.coli* in colony forming units (CFU)/100 ml of sample. The *E. coli* isolated by this method were verified on EMB agar and further by the presence of the *mdh* gene. Four *E. coli* isolates were stored at -70°C from each sample until ready for use. For ESBL isolation one set of filters from each site was extracted with PBS as described below.

### Antibiotic Susceptibility Testing

Colonies grown on Mueller Hinton (MH) agar were used in antibiotic susceptibility testing by the Disk Diffusion method according to Clinical and Laboratory Standards Institute guidelines (CLSI) (ClSI, 2012). The CLSI clinical breakpoints for an antibiotic toward enterobactericiae were used to assign isolates sensitive or resistant status. Altogether 98 isolates from the fecal samples and 184 isolates from the water samples were analyzed. Thirteen antibiotics were tested: ampicillin (AMP or A) 10 μg, amoxicillin-clavulanic acid (AMC) 20 μg, ceftazidime (CAZ) 30 μg, ceftiofur (XNL) 30 μg, tetracycline (T or TE) 30 μg, ciprofloxacin (CIP) 5 μg, enrofloxacin (ENO) 5 μg, chloramphenicol (C) 30 μg, streptomycin (S) 10 μg, spectinomycin (SPT), sulfamethaoxazole/trimethoprim (SXT) 25 μg, nalidixic acid (NA) 30 μg, and neomycin (N) 5 μg. For some of the isolates (pre and post rain) gentamycin (G) 10 μg and kanamycin (K) 30 μg were also evaluated.

### ESBL Selection

Filters obtained from water samples were washed with 300 μl of PBS and the washings were plated onto three MacConkey agar (MCA) plates supplemented with 4 μg/ml Cefotaxime and incubated overnight at 37°C (Durso et al., 2016). Pink colonies obtained were further verified on EMB agar for confirmation as *E. coli*, as described above. Initially CTX was added at a concentration of 1 μg/ ml on the plates, but most of the isolates turned out to be false positives since they failed to regrow on these plates. In addition, all *E. coli* isolates from mColiBlue filters that tested resistant to AMP and CAZ but were susceptible to AMC in disk diffusion assays were further evaluated for ESBL presence by the double disc method (DDST) originally described by Jarlier et al. (1988), with slight modifications. Briefly, a disk containing amoxicillin/Clavulanic acid (AMC) was placed in the center of a MH agar plate spread with the test isolate. At 20 mm apart (center to center) from the AMC disk ceftriaxone (CRO), cefotaxime or ceftazidime were placed on three sides. For several of the isolates Cefoxitin (FOX) was included on a 4th side. The test was considered positive if, after 24-h incubation at 37°C, the zone of inhibition between one or more of the disks was enhanced.

Fecal samples were plated directly on MCA + Cefotaxime plates and pink colonies were saved as putative ESBL containing *E. coli*. They were further tested and confirmed as above. In addition isolates obtained on EMB agar that tested resistant to AMP and CAZ but were susceptible to AMC, were further tested for ESBL phenotype. We also tested for the presence of *bla*_ctx-M_ gene in all water and fecal isolates that were resistant to AMP, CAZ/CTX as well as AMC, as described below.

All procedures were conducted under strict biosafety guidelines laid out by University of Washington Environmental Health and Safety office^3^.

### DNA Isolation and PCR

A 1–2 mm size colony from an overnight culture plate was suspended in 10 μL of Prepman Ultra Sample Preparation Reagent (Life Technologies, Foster City, CA, United States). Alternatively, 1 mL of an overnight culture broth of an isolate was centrifuged at 10,000 *g* for 5 min. The supernatant was removed, and the pellet was re-suspended in 200 μL of Prepman Ultra Sample buffer. In either case, the suspensions were heated at 95°C for 10 min, cooled, and centrifuged at 10,000 *g* for 2 min. Two microliters of the supernatant was directly used in a 20 μL PCR reaction. The supernatants were stored at 4°C if they were to be used within the week otherwise at -20°C. Extracts stored at -20°C performed as well as a fresh preparation in a qPCR or PCR reaction, 20 months later (data not shown).

### Antibiotic Resistance Gene Detection

All isolates that showed antibiotic resistance by phenotypic methods were tested for the respective genetic determinant. Strains that showed ESBL phenotype by the double disc method were tested for *bla*_ctx-M_ *bla*_shv_ and *bla*_tem_ by a qPCR method (Birkett et al., 2007; Angeletti et al., 2013) cefotaxime and/or ceftazidime resistant isolates that were also resistant to AMC were tested for the *bla*_cmy-2_ gene (Alali et al., 2009) as well as *bla*_ctx-M_. The later was tested to eliminate the possibility of an ESBL carrying isolate being missed, by the phenotypic method. For sequencing we used 453–510 bp PCR products obtained by primers Cottell CTX M- F 5′-CCG CTG CCG GTY TTA TC-3′ and Cottell CTX-M R-5′-ATG TGC AGY ACC AGT AA-3′ described earlier (Cottell et al., 2013). We also used another PCR product of 554 bp obtained with forward primer 5′ATG TGC AGY ACC AGT AAR GTK ATG GC-3′ and reverse primer 5′TGG GTR AAR TAR GTS ACC AGA AYS AGC GG-3′ (Hedman et al., 2019). The last set of primers allowed us to distinguish between *bla*_ctx-M27_ and *bla*_ctx-M14_. Tetracycline resistance genes were measured by the method of Ng et al for *tet* (A), *tet* (B), *tet* (C), *tet* (D), *tet* (E ),*tet* (G), *tet* (j), *tet* (k), *tet* (L), *tet* (M), *tet* (O), *tet* (Q), *tet*(S), *tet* (X) (Ng et al., 2001). Additionally qPCR assays were also used for rapid detection of *tet* (M) *and tet* (W) as described earlier (Walsh et al., 2011) Streptomycin resistance was measured by testing for *strA*, *strB*, and *aadA* (Walsh et al., 2011). All qPCR reactions were performed in a Mini-opticon icycler (BioRad). For SYBR green PCR, iTaq^TM^ Universal SYBR green mastermix and for TaqMan^TM^ PCR, iTaq^TM^ Universal Probes Supermix (Bio-rad, Hercules, CA, United States) was used. The cycling parameters for Taqman qPCR was as follows: 1 cycle at 95°C for 10 min, followed by 40 cycles of 15 s at 95°C, 30 s at 58°C, and 30 s at 72°C, with a final cycle of 5 min at 72°C. For tetracycline resistance genes controls were obtained from Dr. Lisa Durso, USDA, NE, United States (Durso et al., 2016) and Dr. Marilyn Roberts (University of Washington). A D-block synthesized by IDT (IDT Inc.) that contained the sequences of the *bla*_ctx-M1_, *bla*_ctx-M2_, and *bla*_ctx-M9_ PCR products as described in Birkett et al. (2007) was used as control for *bla*_ctx-M_ in the initial TaqMan^TM^ PCR. *bla*_ctx-M_ isolates identified thus were then used as positive controls for the other regular PCR reactions. *bla_shv_*, *bla_tem_*, *strA*, *strB*, and *aadA* controls were developed in house from strains that tested positive by PCR and subsequent sequencing. The sequences obtained for *bla*_ctx-M_ gene from the different isolates have been deposited in the GenBank and their accession numbers are: MK78174 to MK781784.

#### Grouping Isolates Based on *mdh* Gene Sequence and MLST Studies

A 825 bp region of the *mdh* gene was amplified and sequenced for several feces and water isolates using the published primers: mdhF: 5′TGAAAGTCGCAGTCCTCGG-3′ and mdhR 5′-TCCACGCCGTTTTTACCC-3′ as described before (Ivanetich et al., 2006). A 282 bp region from this was trimmed, aligned and a phylogenetic tree obtained using the Maximum Likelihood method. Epidemiological relatedness of the isolates was tested using seven *E. coli* housekeeping genes, utilizing MLST. MLST was performed according to the methods specified at the MLST website http://enterobase.warwick.ac.uk/species/index/ecol. The PCR products from the seven housekeeping genes were sequenced using the same primers used to generate the fragments. Sanger sequencing was performed by Eurofins Genomics (Louisville, KY, United States). *E. coli* STs were assigned using the above databases as well as that developed by Keith Jolley [33], at University of Oxford Site: https://pubmlst.org/bigsdb?db=pubmlst_mlst_seqdef\&page=profiles.

#### Phylogenetic Studies

The quadruplex PCR method of Clermont et al. (2013) was used to assign the *E. coli* isolates to one of the eight phylo groups. After initial placement into groups, based on the results of the quadruplex, strains belonging to phylo-groups A and C or D and E were further identified by using C and E specific primer sets, as per Clermont et al. (2013).

#### Statistical Analysis

One sided proportional *Z* test was used to identify significant differences between count data which is represented as percentages, such as percent antibiotic resistant and percent presence of a phylo-group. The *P* values corresponding to the differences are reported in the tables below the graphs.

## Results

### *E. coli* Loading in the Wetland Roost Area

**FIGURE 2:**
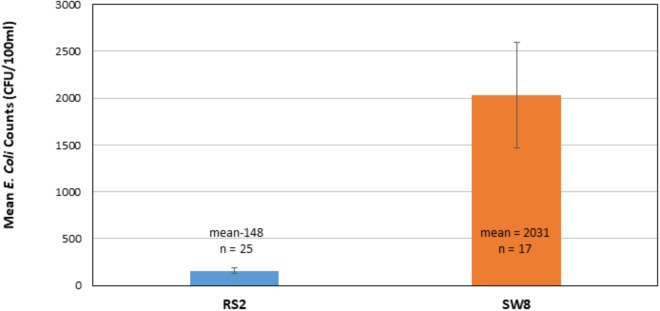
Comparison of mean *E. coli* counts in runoff as it enters (RS2) and leaves the wetland roost zone (SW8). The mean of counts in CFUs, determined 25 times between 2014–2017 at RS2 and 17 times at SW8, is shown. Triplicate samples were collected at each site each time. The error bars represent one standard deviation around the mean for the respective data sets.

Total number of *E. coli* in CFU/100 ml was determined at RS2 site where runoff water from the campus enters the wetland roost area and at the SW8 site where the water exits the roost area, flowing into North Creek (Figure 1, 2). Thus, the number of isolates collected at the RS2 site indicate collection from an area not directly influenced by the crow roost, while SW8 is an area under the direct influence of crows. (Figure 1) The apparent impact of the short journey through the roost zone on the runoff as it flowed from the RS2 site to the SW8 site was an order of magnitude increase in the average *E. coli* count (Figure 2).

### Antibiotic Susceptibility of Crow and Water Isolates

**FIGURE 3:**
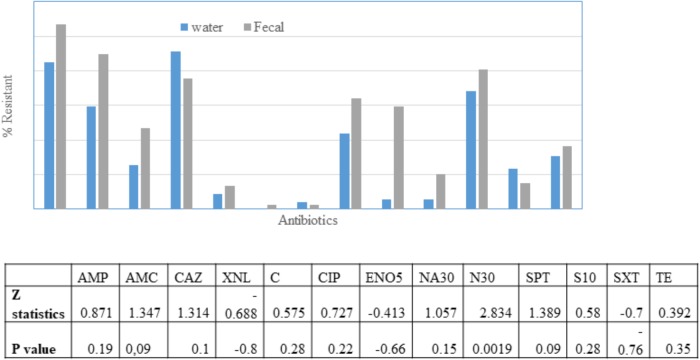
Percentage of *E. coli* in water (*n* = 49) and fecal (*n* = 98) isolates showing non-susceptibility to 13 selected antimicrobials. AMC, amoxicillin/clavulanic acid; AMP, ampicillin; XNL, ceftiofur; C, chloramphenicol; CAZ, Ceftazidime; CIP, ciprofloxacin; ENO, Enrofloxacin; NA, Nalidixic acid; N, Neomycin; STR, streptomycin; SPT, spectinomycin; TE, tetracycline; SXT, trimethoprim/sulfamethoxazole. Table indicates significant difference in antibiotic resistance between water and fecal isolates for 10 antibiotics according to *Z*-test.

The fecal *E. coli* isolated in 2014–2015, were compared with *E. coli* water isolates from the same period for their susceptibilities against 13 antibiotics. 65 and 70% of the isolates from water and crow fecal samples, respectively, were resistant to one or more antibiotics. Ampicillin resistance was the most prevalent, followed by Amoxicillin Clavulanic acid (Figure 3). Multiple drug resistance (three or more of different classes) was found in 40% of the water isolates as well as the crow fecal isolates. Resistance to four antibiotics was most common in water isolates (20%), while among fecal isolates resistance to 4–5 antibiotics was more common (12%). Six fecal isolates showed resistance to seven antibiotics (Table 1). Overall the wetland water isolates showed a similar pattern of susceptibility as that of the fecal isolates for 12 of the 13 antibiotics tested at p value 5% or less (Figure 2). Neomycin was the only antibiotic against which the resistance was significantly different between the water and fecal isolates (*p* ≤ 0.0019), with that in fecal being higher. Among the *tet* and *str* genes tested, *tet* (A), *tet* (B), or *tet* (M) were the genes responsible for >95% of isolates to show the resistance phenotype, while *strA* and/or *strB* was responsible for streptomycin resistance phenotype. *aadA* was detected in a couple of isolates together with *strB. tet* (C) along with tet (D) was present in one fecal isolate. *tet* (M) was usually present with *tet* (A) (15 isolates). Two isolates had *tet* (A), *tet*(B) and *tet* (M) while *tet* (A) and *tet*(B) co-occurred in six isolates. For sulfamethaoxazole/trimethoprim (SXT) resistance the *sul1* gene was tested and it was present in 100% of the isolates that showed the phenotype.

**Table 1:** Percentage of water (*n* = 49) and fecal (*n* = 98) isolates resistant to one or more antibiotics.

No. of Antibiotics	Water (%)	Fecal (%)
0	17 (34)	29 (29.6)
1	8 (16.3)	17 (17.3)
2	3 (6.12)	8 (8.16)
3	2 (4.08)	5 (5.1)
4	10 (20.4)	12 (12.2)
5	5 (10.2)	12 (12.2)
6	4 (8.16)	9 (9.18)
7	0 (0)	6 (6.12)
Multidrug resistant	19 (39.58)	39 (39.8)
(≥3 classes)


#### Antibiotic Susceptibility of *E. coli* Isolates Before (No Rain) and After Rainfall (Rain)

**FIGURE 4:**
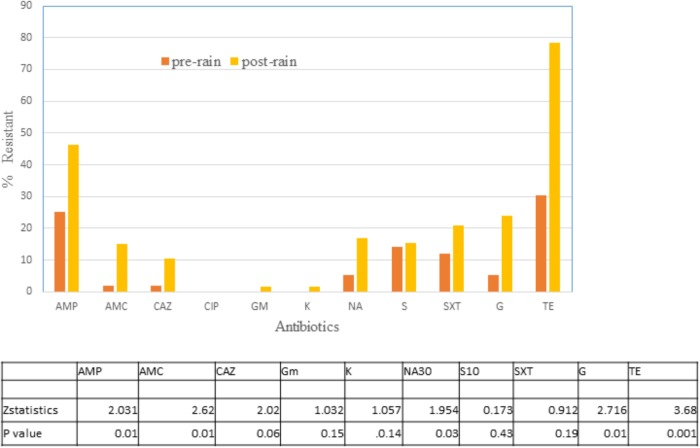
Percentage of *E. coli* isolates in water on no rain days (*n* = 62) and post-rain days (*n* = 63) showing non-susceptibility to 11 selected antimicrobials. AMC, amoxicillin/clavulanic acid; AMP, ampicillin; C, chloramphenicol; CAZ, Ceftazidime; CIP, ciprofloxacin; GM, Gentamycin; K, Kanamycin; NA, Nalidixic acid; STR, streptomycin; TE, tetracycline; SXT, trimethoprim/sulfamethoxazole. Table indicates significant difference in antibiotic resistance between no rain and rain days by *Z* test of proportionality.

Altogether 65 isolates from no rain and 67 from rain days were tested for their susceptibility to 11 antibiotics (Figure 4). There was a significant difference in resistance to TE, AMP, AMC, NA, and gentamycin with rain days demonstrating a higher level of resistance to these antibiotics. No resistance was observed to Ciprofloxacin, and only one isolate each were resistant to gentamycin and kanamycin post rain. For the remaining three antibiotics the difference was not as significant at *p* < 0.05.

#### ESBL and Beta Lactamase *(ampC*) Containing Isolates

Only two ESBL containing *E. coli* were isolated from the water samples collected between 9/17/14 and 4/05/2015, and one more from collections made between 2016–2017 (Table 2). These isolates were obtained initially on m-ColiBlue24 broth and based on their antibiotic profile were plated on MCA+ Cefotaxime. Among the fecal isolates, 7 of the 98 (7.1%) isolates carried ESBL. Except for one, all the fecal isolates were obtained non-selectively on EMB agar for *E. coli*. Since they were ampicillin resistant but AMC susceptible, they were further tested and purified on MCA + CTX and subjected to ESBL verification. Two additional isolates had *bla*_cmy-2_ and *bla*_ctx-m_ and thus 9 of 98 (8.9%) can be considered as ESBL *E. coli*. All ESBL isolates were multi drug resistant with resistance to at least Amp, Caz/Ctx, S, SXT, TE. The *bla*_cmy-2_ gene was present in 16 of 98 (16.3%) fecal isolates and 9 of 49 (18.36%) water isolates in the collections from 2014 and 2015. All of these isolates were first non-selectively isolated for *E. coli* on EMB agar. AMP, AMC, and ceftifuor resistance indicated testing for *bla*_cmy-2_. Seven of the 16 *bla*_cmy-2_ containing isolates were MDR in the fecal isolates. *bla*_shv_ co-occurred with *bla*_cmy-2_ in one instance and with *bla*_tem_ in two instances. For *bla*_tem_ a 189 bp sequence was obtained that had 100% homology with classA ESBL – TEM1, while for bla_shv_, a 193 bp sequence was obtained that had 100% homology to ESBLs – SHV12, SHV-61, SHV-5.

**Table 2:** Sequence type, antibiotic genetic determinant, and phylo-group of fecal and water isolates that had *bla*_ctx-M_ or *bla*_CMY -2_, *bla*_tem_ or *bla*_shv_ genes.

Strain	Round	ST	ESBL phenotype	Resistance Phenotype	AR Genes	Phylo-grou
Fecal						
F4.1	Rl	ST 2614	ND	AMP-AMC-CTX	*bla*_tem_	B2
F7.1	Rl	ST5914	Negative	AMP-AMC-CF-N	*bla*_tem_	Bl
F2.7	Rl	ST8371	Negative	AMP-AMC	*bla*_tem_	D
F11.l	R2	ST131	CAZ,CTX,CRO, FOX, CF	AMP-CTX-CF-XNL-S-SXT-T	*bla*_ctx_, *strA*, *sul1*, *tet* (A), *tet*(M)	B2
F11.2	R2	ST131	CAZ,CTX,CRO, FOX, CF	AMP-CAZ-CF-XNL-S-SXT-T	*bla*_ctx_, *strA*, *str* B, *sul1*, *tet*(A), *tet* (B), *tet* (M)	B2
F13.1	R2	ST131	CAZ,CTX,CRO, FOX, CF	AMP-CAZ-CF-XNL-NA-S-SPT-SXT-T	*bla*_ctx_, *str* A, *str* B, *sul1*, *tet*(A)	B2
F13.2	R2	ST131	CAZ,CTX,CRO, FOX, CF	AMP-CAZ-CF-XNL-S-SPT-SXT-T	*bla*_ctx_, *str* A, *str* B, *sul1*, *tet*(A), *tet* (M)	B2
F14.1^∗^	R2	ST 58	ND	No resistance	No resistance	Bl
F15.2	R2	ST131	CAZ,CTX,CRO, FOX, CF	AMP-CAZ-XNL-N–S-SXT-SPT-T	*bla*_ctx-M_, *str* A, *str* B, *sul1*, *tet* (A), *tet* (M), *tetB*	B2
F16.2	R2	ST131	CAZ,CTX,CRO, FOX, CF	AMP-CAZ-CF-XNL-NA-SXT-T	*bla*_ctx-M_, *bla*_CMY -2_, *str* A, *str* B, *sul1*, *tet*(A),	B2
F20.3^∗∗^	R2	ST68	CAZ,CTX,CRO, FOX,CF	AMP-AMC-CAZ-XNL-N-S-T	*bla*_ctx_, *bla*_CMY -2_, *bla*_shv_, *str* A, *str* B, tet(A)	F
F27.2^∗∗^	R2	ST68	CAZ,CTX,CRO, FOX, CF	AMP-AMC-CAZ-XNL-T	*bla*_ctx_, *bla*_CMY -2_, *bla*_tem_, tet (A) *tet* (M)	F
F31.1	R2	unknown	Negative	AMP-AMC-CTX-XNL-	*bla_CMY -2__ strB*, *tet(B)*,	B2
F32.1	R2	ST10	Negative	AMP-CAZ-XNL-C-NA-S-T	*bla*_CMY -2_, *str* A, *tet* (A)	A
F34.2	R3	ST7348	Negative	AMP-AMC-XNL-C	*Bla_CMY -2_ tet (M)*	Bl
F35.2	R3	ST2541	CAZ,CTX,CRO	AMP-CAZ	*bla*_ctx_	A
F 42.2	R4	Unknown	Negative	AMP-AMC-CAZ-XNL-NA-N-S	*bla_CMY -2_ strB*	Bl
F43.1	R4	ST7207	Negative	AMP-AMC-XNL-C-NA-N-S-T	*bla_CMY -2_ strB*, *tet(B)*, *tet(M)*	A
F44.2	R4	Unknown	Negative	AMP-CAZ-XNL-NA-N	*bla*_CMY_	E
F46.2	R4	ST357	Negative	AMP-CAZ-NA-N-S-T	*bla*_CMY -2_ *strB*, *tet(B)*	B2
F47.2	R4	ST58	Negative	AMP-AMC-XNL-ENO-NA-N-S	*bla*_CMY -2_, *bla*_tem_, *str* A	Bl
F48.1	R4	ST3727	Negative	AMP-AMC-XNL-NA-N	*bla*_CMY -2_	C
F52.2	R5	ST195	Negative	AMP-AMC-CAZ-C	*bla*_CMY -2_	C
Water						
NC5.1ctx	R2	ST131	CAZ,CTX,CRO, FOX	AMP-CAZ-XNL-S-SXT–T	*bla*_ctx_, *str* B, *sul1*, *tet*(A), *tet* (M)	B2
SW2.3 ctx	R2	ST1625	Negative	AMP-AMC-CTX-C-AZ-XNL	*bla*_CMY -2_	Bl
NC5.3	R3	ST2721	Negative	A-AMC-XNL-CF-S-T	*bla*_CMY -2_ *str* B, *tet* (A)	Cryptic Clade III,IV,V
NC6.2^∗^	R3	ST58	ND	No resistance	No resistance	Bl
NC 6.7	R3	ST10	Negative	AMP-CAZ-XNL-C-NA-S-T	*bla*_CMY -2_*bla*_shv_, *strB*, *tet(B)*	A
SW2.4	R3	ST83	Negative	AMP-AMC-CAZ-XNL-NA-SPT-S-T	*bla*_cmy-2_*strB*	B2
NC5.2	R3	ST1204	Negative	AMP-AMC-XNL-CF-S-T	*bla*_CMY -2_ *bla*_shv_, *str* A,*strB*, *tet* (M)	D
RP3.1ctx	R3	Unknown	CAZ-CTX-CRO, FOX	AMP-CTX-XNL-NA-SPT-S-SXT-T	*bla*_ctx_, *aadA*,*sull*, *tet(M)*	E
RP3.1 (Box)	R3	ST5463	Negative	AMP-CTX-XNL-CF-NA-SPT-S-SXT-T	*bla*_shv_, *str* A, *sul1*, *tet* (B)	E
RP3.2^∗^	R4	ST58	Negative	AMP-AMC-CTX-XNL-ENO-NA-N	*bla*_CMY -2_	Bl
RP3.5 (CAT)	R4	ST469	Negative	AMP-XNL-CF-NA-S	*bla*_tem_, *strA*	Bl
SW2.2	R4	ST1065	Negative	AMC-CTX-CF-S	*bla*, *strA*,*strB*	B2
SW2.4	R5	ST1850	Negative	AMP-AMC-CAZ-CF-XNL-S-SXT-C	*bla_CMY_._2_ strB*, *sull*	A
RP3.6	R5	Unknown	Negative	CF-XNL	*bla*_CMY_	C
RSI.3	6/16/2017	Unknown	CAZ-CTX-CRO-CF	AMP-CAZ-CF-XNL-S-SXT-C	*bla*_ctx_, *tet*(B), *str* A *sul1*	E
RS2.1 Mcoli	4/6/2018	ST297	CAZ-CTX-CRO-FOX-CF	AMP-AMC-CAZ-CF-CRO	*bla*_ctx_	B1


*^∗^Selected because they had same *mdh* (282 bp) sequence (Ivanetich et al., 2006). AR, antibiotic resistance; ST, Sequence Type. R1, R2, R3, R4, R5 are collection dates from 8/20/14, 9/17/14, 1/21/15, 2/27/15, 4/5/15, respectively. RS2.1 Mcoli was an isolate with the *bla* ctx gene that was found in the summer of 2018 (June–August) from two rounds of sampling that screened 15 isolates. ^∗∗^Indicate isolates that had both bla ctx-M and bla cmy-2 genes*.

### *E. coli* Sequence Types in Water and Fecal Samples

A total of 39 isolates, 23 fecal and 16 water, were selected for MLST. This was based on presence of *bla*_ctx-M_, *bla*_cmy-_*_2_*, *bla*_tem_, or *bla*_shv_ gene. Care was taken to see that there were representative isolates from different collection dates, both from water and fecal. A phylogenetic tree based on presence of 282 bp of the *mdh* gene alone was obtained for 30 crow fecal and 29 water isolates from 2014–2015, Supplementary Figure S1 as described earlier (Ivanetich et al., 2006). The isolates were randomly chosen, however, isolates from each collection were included for determination of *mdh* presence. Eight clusters (a cluster was formed if three isolates had identical 282 bp region) were obtained. Where water and fecal isolates clustered together, a bigger region of the *mdh* gene that encompassed the 452 bp region, that is used for MLST analysis, was aligned and if the same allele was obtained then sequencing of the remaining six housekeeping genes was undertaken. For example, F35.1 had *mdh* gene corresponding to allele 16, while the ESBL isolates in this cluster had an *mdh* gene with allele 36, and thus F35.1 was not subjected to MLST. In this manner F14.1 and NC6.2 (R2) were selected and identified as ST58 and F32.1 and NC6.7 (R2) as ST10. Two isolates from the fecal samples F11 and F13 were analyzed because their antibiotic resistance phenotype was a little different although they both had the *bla*_ctx-M-27_ gene.

Multilocus Sequence Typing analysis showed high diversity in the sequence types obtained from the different collection dates. 13 different STs were obtained for the fecal isolates and 10 for the water isolates. STs of 4 of the 39 isolates could not be determined (Table 2). Within one collection date, although there was genetic diversity, several identical STs were obtained within the fecal isolates. Thus 6 of 16 isolates from the 9/15/14 collection belonged to ST131, while 2 of 12 from 11/10/14 collection belonged to ST68. All ST131 isolates had the bla_ctx-M_ gene and sequencing of the gene showed them to be bla_ctx-M-27_.

When STs from water and fecal isolates were compared, in three instances a common ST was found in the water and fecal. Thus one ST131 isolate, NC 5.1 ctx, found in a water sample from NC5 site (Figure 1) on 9/14/15, was found in several (six) fecal isolates from the same date (Table 2). Fecal isolate F32.1 from 1/12/15 had ST10, a ST which was also found in a water sample NC 6.7 from site NC6 on the same date. Similarly, ST58 was found in a fecal isolate F47.2 as well as water isolate RP3.2, both isolated on 2/27/15.

**FIGURE 5:**
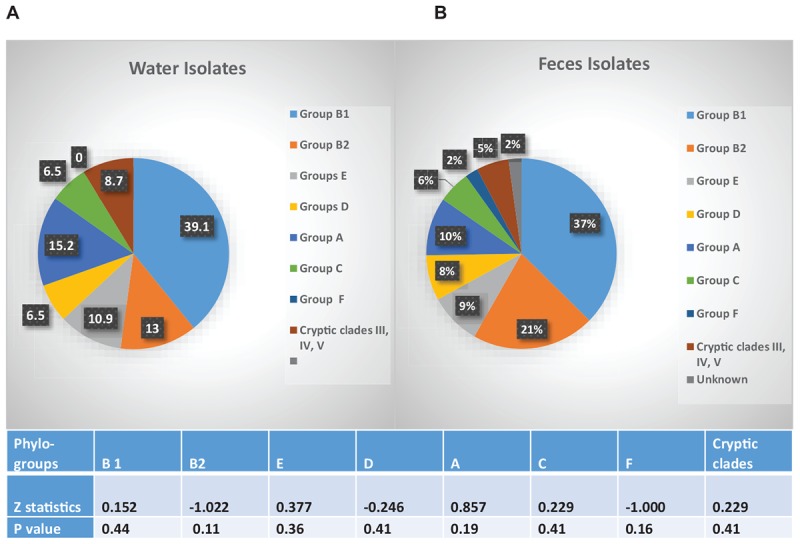
Phylo-grouping of the Isolates. Percentage of *E. coli* isolates from water (*n* = 46) and feces (*n* = 92) belonging to each phylo-group. Different groups are represented by different colors. Among the water isolates there were no unknowns. Table indicates significant difference in presence of each phylo-group, between water and fecal samples.

The fecal and water isolates were phylo-typed by the method of Clermont et al. (2013). The largest percentage of *E. coli* isolates from both crow fecal (*n* = 91) and surface water (*n* = 46) samples belonged to the non-pathogenic, commensal phylo-group B1, followed by the pathogenic B2 group (Figure 5). Statistical analysis revealed no significant difference in the presence of any of the phylo-groups across the water and fecal isolates (*p* > 0.05). Although the B2 and D phylo-groups, the two groups where most of the ExPEC strains are expected to belong, have a slightly more representation among the fecal isolates, the numbers are not statistically significant.

## Discussion

Several studies have reported that the environment imposes its own selection on the population of *E.coli* following fecal deposition from its primary habitat within the intestine of animals (Gordon et al., 2002; Bergholz et al., 2011; Jang et al., 2017). As a result a new genomic diversity may develop with species that are stress tolerant and are able to adapt locally to that particular matrix being amplified and over represented. To what extent this will happen is a subject of much debate and study, nonetheless, it is generally agreed that fecal deposition is the major predictor of the population structure of the matrix (Bergholz et al., 2011; Jang et al., 2017). Thus, while there were differences in the genetic diversity of the *E. coli* isolated from the crow fecal isolates in our wetland, from the limited sequence typing we performed, the finding of similar antibiotic resistance pattern between the water and crow isolates is not unexpected.

The fecal population showed no significant difference in the overall resistance to twelve of the 13 antibiotics tested, when compared to that of the water population. Some of the drug resistance genetic determinants may be on mobile genetic elements, e.g., plasmids were isolated from F20.3, F46.1, and RP3.5 ctx, F15.2 (results not shown) and these have the ability to be transmitted to the indigenous bacteria in the wetland (Aminov, 2011; Wellington et al., 2013). The number of isolates resistant to at least one antibiotic in the crows (70%) and water (65%) was high in our study. In 97% of our isolates we were able to find the corresponding genetic determinant of the phenotypic antibiotic resistance displayed by an isolate. The distribution of isolates based on their phylo-group, proved to be similar between the fecal and water samples, providing additional support that crow fecal deposition drives the distribution of the strains in water. The high proportion of B1 phylo-group (37% in fecal and 39% in water) in our isolates agrees well with one other recent study which found high percentages of the commensal *E. coli* phylo-group B1 in the fecal (38%) and soil (40%) samples collected in a recreational meadow (Bergholz et al., 2011). They correlated phylo-group B1 *E. coli* with the presence of feces from wild and domestic animals. In our study, however, presence of the B2 phylo-group cannot be ignored because of their potential to cause disease. 21 and 13% of the fecal and water isolates, respectively, belonged to the B2 phylo-group, which is expected to contain the majority of the extra intestinal pathogenic *E. coli* (ExPEC) strains and may come from a human source (Picard et al., 1999; Tenaillon et al., 2010). The D group which contains some ExPEC strains was also represented in the fecal and water samples. Further characterization of the virulence genes from these isolates are in progress.

We found a predominance of *bla*_cmy-2_ gene in the AmpC phenotype in the crow (16.8%) and water (18.36%) isolates. *bla*_cmy-2_ has been shown to be the most common plasmid borne beta lactamase in human, animal, and environmental bacterial isolates, and that includes large corvids in United States and Canada (Pitout et al., 2007; Mataseje et al., 2010; Folster et al., 2011; Martin et al., 2012; Jamborova et al., 2017, 2018). In a recent report 18.7% of Corvids from Canada were shown to carry the *bla*_cmy-2_, which was substantially more than that reported from Corvids from European countries (4.4%). The authors suggested a difference in population dynamics of antimicrobial resistance in *E. coli* between the two continents. While our sample size and survey is small, this may be true for the United States as well, especially since another report on *E. coli* isolated from different cities of the United States from the same species of corvid as ours, viz., *C. brachyrhynchos*, described 15–19% presence of *bla*_cmy-2_ (Jamborova et al., 2017). *bla*_cmy-2_ is not very commonly isolated among clinical isolates in the United States (Castanheira et al., 2013). The *bla*_cmy-2_ isolates in this study could have come from any number of sources. MLST analysis revealed a genetic diversity within and between the fecal and water *E. coli* isolates possessing the *bla*_cmy-2_. Sequence types frequently isolated from companion animals as well as livestock and farm animals, besides humans, were found in these isolates. Thus, ST7207, ST5914, ST2721, ST2541, ST1204 found in our study were shown to have been isolated from livestock and water sources^4^. Agricultural and rural lands are abundant in the nearby Snohomish County, WA and it is possible that the crows acquired some of these strains from the farm animals that live there. Other *bla*_cmy-2_ possessing sequence types found in this study, viz., ST58, ST 83, ST357, which have been shown to belong to Avian Pathogenic *E. coli* (APEC) group, have been reported to be found in birds including crows, poultry, companion animals, as well as humans (Dissanayake et al., 2014; Jamborova et al., 2017). In humans they been described as ExPEC strains capable of causing urinary tract infections among other infections, but can be present as non-pathogens as well. Two ST58 strains in our study had no virulence genes or antibiotic resistance genes. Two other fecal isolates, ST8371 and ST2614, have previously been reported to be isolated only from humans^4^.

The most unexpected finding was the presence of an isolate (NC5.3 ctx) belonging to ST131 from the North Creek site within the roosting area of the wetland. ST131, a pandemic clone, has been shown to be responsible for severe extra intestinal infections in animals and humans, besides being MDR (Johnson et al., 2010). In the United States it was first reported in 2007 (Johnson et al., 2010). The wetland is situated within the UWB/CC campus which has a maximum population of 6000 students and thus is not crowded. The campus septic wastewater is entirely piped offsite for treatment and there are no septic systems or porta-potties on campus. However, North Creek originates in the highly urbanized City of Everett flowing 12.6 miles southward through suburban areas of the cities of Mill Creek and Bothell before reaching the UWB/CC campus, passing the roost area, and draining into the Sammamish River. There are many houses with septic systems in the North Creek drainage basin (City of Bothell 2019) and the creek has received raw sewage discharges multiple times between 2012 and 2018 during peak rainfall events (King County, 2014). Overbank flooding from North Creek did not occur during sampling, so North Creek water did not impact any of the wetland water samples. Nor were water samples collected during or shortly after the sewage overflow events (eight between 11/24/16 and 3/18/17) from upstream manhole 54 of the North Creek Interceptor sewer line. Isolate NC5.3 ctx had an antibiotic resistance phenotype that matched with the fecal isolate F11.1 which also was ST131. It is tempting to speculate that the water ST131 came from one of the crows. The omnivorous feeding habit of the crows, together with their synanthropic behavior may very well allow them to be colonized by MDR bacteria. This has been shown in other studies as well (Jamborova et al., 2017). In addition, these North American crows can fly as far as 40 miles per day away from their roosting site in non-breeding seasons (Link, 2005) to acquire food, and these may include agricultural and rural areas as well (Roberts et al., 2016). All of the ST 131 isolates belonged to the phylo-group B2, indicating the isolates may be virulent strains.

By grouping the isolates based on the *mdh* gene (Supplementary Figure S1) and performing MLST on selected isolates within a cluster, we were able to find two more sequence types from the water that matched with those of crows and both were collected on the same respective dates as the fecal isolates. The phylo-group and antibiotic resistance phenotype matched in both cases. Both STs have been reported to be found from crows as well as humans. Analysis by techniques such as Pulsed-field gel electrophoresis or repetitive sequence-based PCR or Whole Genome sequencing can further firmly establish the clonal relationship of these isolates. Interestingly, the ST131 strain found in both fecal and water samples in September, 2014 was not found again in subsequent isolations from 2014, 2015, 2016, or 2017. This was also true for the other two isolates with matching STs. Only one ST58 (F14.1) found in September, 2014, was seen in water collection of February, 2015 (RP3.2). Their AR phenotype matched, but the exact clonal relationship needs to be confirmed. Thus, it appears that most of these strains may not be able to survive for long in the environment. *E. coli* abundance is known to decline over months in water and soil matrices, although persistent strains may remain (Avery et al., 2004; Vivant et al., 2016). It can be speculated that the isolates are not able to survive in the crow gut either for any length of time, since the crows are known to roost in the same area repeatedly (Link, 2005) and the STs were not recovered in the following months. Further studies are needed to understand how long they persist in the gastrointestinal tracts of the birds. We continued to monitor for ESBL *E. coli* in water through 2016, 2017 and spring 2018 at the roosting sites. We were able to find only two more ESBL containing isolates, one of which belonged to ST297, and for the other we were not able to find a ST, even though we found a matching allele for each of the seven genes in both of the MLST data bases that we used.

Increase in antibiotic resistant *E. coli* in storm water runoff has been reported by Salmore et al. (2006) and increase in ARGs due to storm water loading was recently reported by Garner et al. (2017). Our study also detected additional ARE following rainfall, with tetracycline resistance increasing the most. While the crows deposit the bulk of their feces in the roost area, they gather for short periods each dusk and dawn all over the campus leading to widespread deposition of feces. During dry periods, the crow feces and the bacteria contained within them accumulate on campus. During rain events, these bacteria are mobilized, flowing in the storm water system. It is also possible septic systems within the North Creek watershed overflow during a storm event, contributing additional bacteria. An increase in overall *E. coli* count was also observed at the sampling sites, both within and outside the roost area in response to rain events (Supplementary Figure S2).

## Conclusion

In conclusion, although most of the crow deposited strains may not be able to survive for long in the wetland, there appears to be a constant addition of AR bacteria, and most of them appear to be coming from the crows because the overall pathogenicity and AR pattern of the wetland water isolates were very similar to that of the birds’ fecal isolates over the course of 9 months that they were tested. Regardless, the crows do drink this water and ingest the *E. coli* during their daily visitation to the wetland. They are thus potential vectors for transmission of the multiple drug resistant strains (as well as non-virulent and non-AR ones) to various places during their daytime scavenging activities. They are also partially migratory, with populations moving to more southern latitudes of North America during the winter and thus these strains may be carried even further during the winter months (Verbeek and Caffrey, 2002), posing an overall public health risk. This first report from one of the largest crow roost areas within the state of Washington, highlights the risks that the crows may pose for the spread of antibiotic resistance and the need for remedial measures.

## Author Contributions

KS designed and supervised all experiments and did data analysis, performed some experiments and prepared the manuscript. RT identified sites in the wetland to collect samples, determined coliform and *E. coli* counts and helped with manuscript preparation. TB collected samples, determined AR by phenotypic and genotypic methods, performed MLST and data analysis. MS collected samples, did AR studies, *mdh* sequence based phylogenetic studies, and data analysis. BT did MLST and phylo-grouping studies. YM determined all *tet* genes and *sul1* gene presence. MF determined *str* gene presence, *tem* and *shv* sequences and plasmid isolation. LK did phylo-grouping studies. JL helped with manuscript preparation and some analysis.

## Conflict of Interest Statement

The authors declare that the research was conducted in the absence of any commercial or financial relationships that could be construed as a potential conflict of interest.

## Footnotes

1 http://weather.wsu.edu/index.php?page=station_details\&UNIT_ID=330026

2 https://www.hach.com/asset-get.download-en.jsa?id=7639984023

3 https://www.ehs.washington.edu/

4 http://enterobase.warwick.ac.uk/species/ecoli/search_strains

## Supplementary Material

FIGURE S1 Molecular Phylogenetic analysis of the crow and water isolates based on *mdh* 282 bp region. A 825 bp region of the mdh gene was amplified and sequenced for 32 fecal and 29 water isolates. For Fecal samples 11 and 13, named F11 and F13, respectively, two isolates were sampled. A 282 bp region from this was trimmed, aligned, and a phylogenetic tree was obtained using the Maximum Likelihood method based on the Tamur-Nei model. Eight clusters (at least three isolates with the same sequence) were obtained as marked. The different rounds of collection are denoted as: 8-20-14 (R1) 9-5-15 (R2), 1-21-15 (R3), 2-27-15, (R4), 4-5-15(R5). Accession numbers of the *mdh* sequences deposited in GenBank are: MK564267 to MK564325.

FIGURE S2 Impact of Rain events on total counts of *E. coli*. Total number of *E. coli* in CFUs was determined at three of the sites, RS1, RS2, and SW8 before and after a rainfall event. The number of times (N) this was determined at each site is indicated in the figure.

## References

[B1] Alali W. Q. Scott H. M. Norby B. Gebreyes W. Loneragan G. H. (2009). Quantification of the bla(CMY-2) in feces from beef feedlot cattle administered three different doses of ceftiofur in a longitudinal controlled field trial. *Foodborne Pathog. Dis.* 6 917–924. 10.1089/fpd.2009.0271 19622032

[B2] Aminov R. I. (2011). Horizontal gene exchange in environmental microbiota. *Front. Microbiol.* 2:158 10.3389/fmicb.2011.00158 PMC3145257 21845185

[B3] Angeletti S. Gherardi G. De Florio L. Avola A. Crea F. Riva E. (2013). Real-time polymerase chain reaction with melting analysis of positive blood culture specimens in bloodstream infections: diagnostic value and turnaround time. *New Microbiol.* 36 65–74. 23435817

[B4] Avery S. M. Moore A. Hutchison M. L. (2004). Fate of *Escherichia coli* originating from livestock faeces deposited directly onto pasture. *Lett. Appl. Microbiol.* 38 355–359. 15059203 10.1111/j.1472-765X.2004.01501.x

[B5] Baquero F. Martinez J. L. Canton R. (2008). Antibiotics and antibiotic resistance in water environments. *Curr. Opin. Biotechnol.* 19 260–265. 10.1016/j.copbio.2008.05.006 18534838

[B6] Bergholz P. W. Noar J. D. Buckley D. H. (2011). Environmental patterns are imposed on the population structure of *Escherichia coli* after fecal deposition. *Appl. Environ. Microbiol.* 77 211–219. 10.1128/AEM.01880-10 21075897 PMC3019742

[B7] Birkett C. I. Ludlam H. A. Woodford N. Brown D. F. Brown N. M. Roberts M. T. (2007). Real-time TaqMan PCR for rapid detection and typing of genes encoding CTX-M extended-spectrum beta-lactamases. *J. Med. Microbiol.* 56 52–55. 17172517 10.1099/jmm.0.46909-0

[B8] Castanheira M. Farrell S. E. Deshpande L. M. Mendes R. E. Jones R. N. (2013). Prevalence of beta-lactamase-encoding genes among *Enterobacteriaceae* bacteremia isolates collected in 26 U.S. hospitals: report from the SENTRY Antimicrobial Surveillance Program (2010). *Antimicrob. Agents Chemother.* 57 3012–3020. 10.1128/AAC.02252-12 23587957 PMC3697373

[B9] Centers for Disease Control and Prevention [CDC] (2013). *Antibiotic Resistance Threats in the United States, 2013.* Washington DC: Center for Disease Control.

[B10] Clermont O. Christenson J. K. Denamur E. Gordon D. M. (2013). The Clermont *Escherichia coli* phylo-typing method revisited: improvement of specificity and detection of new phylo-groups. *Environ. Microbiol. Rep.* 5 58–65. 10.1111/1758-2229.12019 23757131

[B11] ClSI (2012). *Performance Standards for Antibiotic Disk Susceptibility Tests: Approved Standard* 11th Edn. Wayne, PA: CLSI.

[B12] Coleman B. L. Louie M. Salvadori M. I. McEwen S. A. Neumann N. Sibley K. (2013). Contamination of canadian private drinking water sources with antimicrobial resistant *Escherichia coli*. *Water Res.* 47 3026–3036. 10.1016/j.watres.2013.03.008 23548566

[B13] Cottell J. L. Kanwar N. Castillo-Courtade L. Chalmers G. Scott H. M. Norby B. (2013). blaCTX-M-32 on an IncN plasmid in *Escherichia coli* from beef cattle in the United States. *Antimicrob. Agents Chemother.* 57 1096–1097. 23165469 10.1128/AAC.01750-12 PMC3553738

[B14] Dissanayake D. R. Octavia S. Lan R. (2014). Population structure and virulence content of avian pathogenic *Escherichia coli* isolated from outbreaks in Sri Lanka. *Vet. Microbiol.* 168 403–412. 10.1016/j.vetmic.2013.11.028 24388626

[B15] Dolejská M. Bierošová B. Kohoutová L. Literák I. Čížek A. (2009). Antibiotic-resistant *Salmonella* and *Escherichia coli* isolates with integrons and extended-spectrum beta-lactamases in surface water and sympatric black-headed gulls. *J. Appl. Microbiol.* 106 1941–1950. 10.1111/j.1365-2672.2009.04155.x 19245407

[B16] Durso L. M. Wedin D. A. Gilley J. E. Miller D. N. Marx D. B. (2016). Assessment of selected antibiotic resistances in ungrazed native nebraska prairie soils. *J. Environ. Qual.* 45 454–462. 10.2134/jeq2015.06.0280 27065391

[B17] Folster J. P. Pecic G. McCullough A. Rickert R. Whichard J. M. (2011). Characterization of bla(CMY)-encoding plasmids among *Salmonella* isolated in the United States in 2007. *Foodborne Pathog. Dis.* 8 1289–1294. 10.1089/fpd.2011.0944 21883005

[B18] Garner E. Benitez R. von Wagoner E. Sawyer R. Schaberg E. Hession W. C. (2017). Stormwater loadings of antibiotic resistance genes in an urban stream. *Water Res.* 123 144–152. 28662396 10.1016/j.watres.2017.06.046

[B19] Gordon D. M. Bauer S. Johnson J. R. (2002). The genetic structure of *Escherichia coli* populations in primary and secondary habitats. *Microbiology* 148 1513–1522. 11988526 10.1099/00221287-148-5-1513

[B20] Guenther S. Ewers C. Wieler L. H. (2011). Extended-spectrum beta-lactamases producing *E. coli* in wildlife, yet another form of environmental pollution?. *Front. Microbiol.* 2:246. 10.3389/fmicb.2011.00246 22203818 PMC3244693

[B21] Hasan B. Olsen B. Alam A. Akter L. Melhus A. (2015). Dissemination of the multidrug-resistant extended-spectrum beta-lactamase-producing *Escherichia coli* O25b-ST131 clone and the role of house crow (*Corvus splendens*) foraging on hospital waste in Bangladesh. *Clin. Microbiol. Infect.* 21 1000.e1–1000.e4. 10.1016/j.cmi.2015.06.016 26115863

[B22] Hedman H. D. Eisenberg J. N. S. Vasco K. A. Blair C. N. Trueba G. Berrocal V. J. (2019). High prevalence of extended-spectrum beta-lactamase CTX-M-producing *Escherichia coli* in small-scale poultry farming in rural ecuador. *Am. J. Trop. Med. Hyg.* 100 374–376. 10.4269/ajtmh.18-0173 30457098 PMC6367627

[B23] Ibekwe A. M. Murinda S. E. DebRoy C. Reddy G. B. (2016). Potential pathogens, antimicrobial patterns and genotypic diversity of *Escherichia coli* isolates in constructed wetlands treating swine wastewater. *FEMS Microbiol. Ecol.* 92:fiw006. 10.1093/femsec/fiw006 26839381

[B24] Ivanetich K. M. Hsu P. H. Wunderlich K. M. Messenger E. Walkup W. G. IV Scott T. M. (2006). Microbial source tracking by DNA sequence analysis of the *Escherichia coli* malate dehydrogenase gene. *J. Microbiol. Methods* 67 507–526. 16973226 10.1016/j.mimet.2006.04.026

[B25] Jamborova I. Dolejska M. Vojtech J. Guenther S. Uricariu R. Drozdowska J. (2015). Plasmid-mediated resistance to cephalosporins and fluoroquinolones in various *Escherichia coli* sequence types isolated from rooks wintering in Europe. *Appl. Environ. Microbiol.* 81 648–657. 10.1128/AEM.02459-14 25381245 PMC4277596

[B26] Jamborova I. Dolejska M. Zurek L. Townsend A. K. Clark A. B. Ellis J. C. (2017). Plasmid-mediated resistance to cephalosporins and quinolones in *Escherichia coli* from American crows in the USA. *Environ. Microbiol.* 19 2025–2036. 10.1111/1462-2920.13722 28276133

[B27] Jamborova I. Janecko N. Halova D. Sedmik J. Mezerova K. Papousek I. (2018). Molecular characterization of plasmid-mediated AmpC beta-lactamase- and extended-spectrum beta-lactamase-producing *Escherichia coli* and *Klebsiella pneumoniae* among corvids (*Corvus brachyrhynchos* and *Corvus corax*) roosting in Canada. *FEMS Microbiol. Ecol.* 94:fiy166. 10.1093/femsec/fiy166 30137290

[B28] Jang J. Hur H. G. Sadowsky M. J. Byappanahalli M. N. Yan T. Ishii S. (2017). Environmental *Escherichia coli*: ecology and public health implications-a review. *J. Appl. Microbiol.* 123 570–581. 10.1111/jam.13468 28383815

[B29] Jarlier V. Nicolas M. H. Fournier G. Philippon A. (1988). Extended broad-spectrum beta-lactamases conferring transferable resistance to newer beta-lactam agents in *Enterobacteriaceae*: hospital prevalence and susceptibility patterns. *Rev. Infect. Dis.* 10 867–878. 3263690 10.1093/clinids/10.4.867

[B30] Johnson J. R. Johnston B. Clabots C. Kuskowski M. A. Castanheira M. (2010). Escherichia coli sequence type ST131 as the major cause of serious multidrug-resistant *E. coli* Infections in the United States. *Clin. Infect. Dis.* 51 286–294. 10.1086/653932 20572763

[B31] Li X. Watanabe N. Xiao C. Harter T. McCowan B. Liu Y. (2014). Antibiotic-resistant *E. coli* in surface water and groundwater in dairy operations in Northern California. *Environ. Monit. Assess.* 186 1253–1260. 10.1007/s10661-013-3454-2 24097011

[B32] Link R. (2005). *Living With Wildlife-Crows.* Washington, DC: Department of Fish & Wildlife.

[B33] Literak I. Vanko R. Dolejska M. Cizek A. Karpiskova R. (2007). Antibiotic resistant *Escherichia coli* and *Salmonella* in Russian rooks (*Corvus frugilegus*) wintering in the Czech Republic. *Lett. Appl. Microbiol.* 45 616–621. 17916127 10.1111/j.1472-765X.2007.02236.x

[B34] Martin L. C. Weir E. K. Poppe C. Reid-Smith R. J. Boerlin P. (2012). Characterization of blaCMY-2 plasmids in *Salmonella* and *Escherichia coli* isolates from food animals in Canada. *Appl. Environ. Microbiol.* 78 1285–1287. 10.1128/AEM.06498-11 22156427 PMC3273016

[B35] Martinez J. L. (2009). Environmental pollution by antibiotics and by antibiotic resistance determinants. *Environ. Pollut.* 157 2893–2902. 10.1016/j.envpol.2009.05.051 19560847

[B36] Mataseje L. F. Baudry P. J. Zhanel G. G. Morck D. W. Read R. R. Louie M. (2010). Comparison of CMY-2 plasmids isolated from human, animal, and environmental *Escherichia coli* and *Salmonella* spp. from Canada. *Diagn. Microbiol. Infect. Dis.* 67 387–391. 10.1016/j.diagmicrobio.2010.02.027 20638610

[B37] Ng L. K. Martin I. Alfa M. Mulvey M. (2001). Multiplex PCR for the detection of tetracycline resistant genes. *Mol. Cell Probes* 15 209–215. 11513555 10.1006/mcpr.2001.0363

[B38] Oravcova V. Zurek L. Townsend A. Clark A. B. Ellis J. C. Cizek A. (2014). American crows as carriers of vancomycin-resistant enterococci with vanA gene. *Environ. Microbiol.* 16 939–949. 10.1111/1462-2920.12213 23919480

[B39] Picard B. Garcia J. S. Gouriou S. Duriez P. Brahimi N. Bingen E. (1999). The link between phylogeny and virulence in *Escherichia coli* extraintestinal infection. *Infect. Immun.* 67 546–553. 9916057 10.1128/iai.67.2.546-553.1999 PMC96353

[B40] Pitout J. D. Gregson D. B. Church D. L. Laupland K. B. (2007). Population-based laboratory surveillance for AmpC beta-lactamase-producing *Escherichia coli*, Calgary. *Emerg. Infect. Dis.* 13 443–448. 17552098 10.3201/eid1303.060447 PMC2725889

[B41] Roberts M. C. No D. B. Marzluff J. M. Delap J. H. Turner R. (2016). Vancomycin resistant *Enterococcus* spp. from crows and their environment in metropolitan Washington State, USA: is there a correlation between VRE positive crows and the environment? *Vet. Microbiol.* 194 48–54. 10.1016/j.vetmic.2016.01.022 26876004

[B42] Rodriguez-Mozaz S. Chamorro S. Marti E. Huerta B. Gros M. Sànchez-Melsió A. (2015). Occurrence of antibiotics and antibiotic resistance genes in hospital and urban wastewaters and their impact on the receiving river. *Water Res.* 69 234–242. 10.1016/j.watres.2014.11.021 25482914

[B43] Salmore A. K. Hollis E. J. McLellan S. L. (2006). Delineation of a chemical and biological signature for stormwater pollution in an urban river. *J. Water Health* 4 247–262. 16813017

[B44] Sen K. Lu J. Mukherjee P. Berglund T. Varughese E. Mukhopadhyay A. K. (2018). Campylobacter jejuni colonization in the crow gut involves many deletions within the cytolethal distending toxin gene cluster. *Appl. Environ. Microbiol.* 84:e01893-17. 10.1128/AEM.01893-17 29330183 PMC5835742

[B45] Tenaillon O. Skurnik D. Picard B. Denamur E. (2010). The population genetics of commensal *Escherichia coli*. *Nat. Rev. Microbiol.* 8 207–217. 10.1038/nrmicro2298 20157339

[B46] van Den Bogaard A. E. London N. Stobberingh E. E. (2000). Antimicrobial resistance in pig faecal samples from the Netherlands (five abattoirs) and Sweden. *J. Antimicrob. Chemother.* 45 663–671. 10797090 10.1093/jac/45.5.663

[B47] Verbeek N. A. Caffrey C. (2002). *American Crow (Corvus brachyrhynchos). The Birds of North America.* Ithaca: Cornell Lab of Orinthology.

[B48] Vivant A. L. Boutin C. Prost-Boucle S. Papias S. Hartmann A. Depret G. (2016). Free water surface constructed wetlands limit the dissemination of extended-spectrum beta-lactamase producing *Escherichia coli* in the natural environment. *Water Res.* 104 178–188. 10.1016/j.watres.2016.08.015 27522634

[B49] Walsh F. Ingenfeld A. Zampicolli M. Hilber-Bodmer M. Frey J. E. Duffy B. (2011). Real-time PCR methods for quantitative monitoring of streptomycin and tetracycline resistance genes in agricultural ecosystems. *J. Microbiol. Methods* 86 150–155. 10.1016/j.mimet.2011.04.011 21549164

[B50] Wellington E. M. Boxall A. B. Cross P. Feil E. J. Gaze W. H. Hawkey P. M. (2013). The role of the natural environment in the emergence of antibiotic resistance in gram-negative bacteria. *Lancet Infect. Dis.* 13 155–165. 10.1016/S1473-3099(12)70317-1 23347633

[B51] WHO (2014). *Antimicrobial Resistance: Global Report on Surveillance 2014.* Geneva: World Health Organization.

