# Supplementary figures and images for: Antibiotic Resistance of E. coli Isolated From a Constructed Wetland Dominated by a Crow Roost, With Emphasis on ESBL and AmpC Containing E. coli
Source: Front Microbiol. 2019 May 15;10:1034. doi: 10.3389/fmicb.2019.01034 (PMC6530415; doi:10.3389/fmicb.2019.01034)

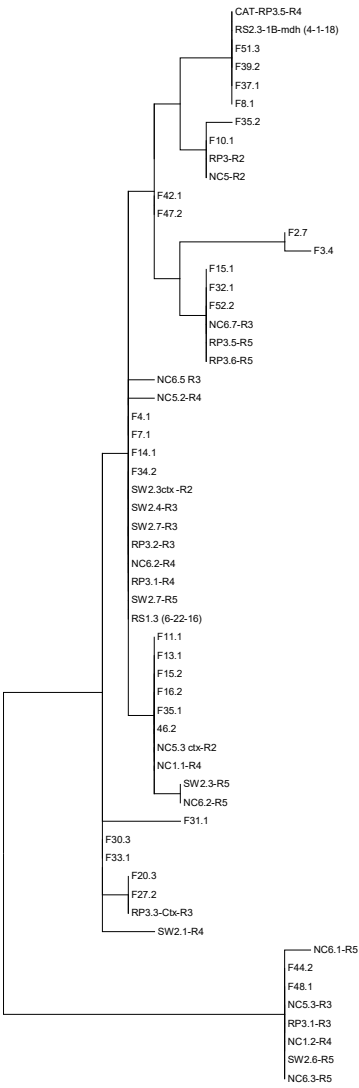

Supplement: FIGURE S1 — Molecular Phylogenetic analysis of the crow and water isolates based on mdh 282 bp region. A 825 bp region of the mdh gene was amplified and sequenced for 32 fecal and 29 water isolates. For Fecal samples 11 and 13, named F11 and F13, respectively, two isolates were sampled. A 282 bp region from this was trimmed, aligned, and a phylogenetic tree was obtained using the Maximum Likelihood method based on the Tamur-Nei model. Eight clusters (at least three isolates with the same sequence) were obtained as marked. The different rounds of collection are denoted as: 8-20-14 (R1) 9-5-15 (R2), 1-21-15 (R3), 2-27-15, (R4), 4-5-15(R5). Accession numbers of the mdh sequences deposited in GenBank are: MK564267 to MK564325. [file Image_1.pdf]

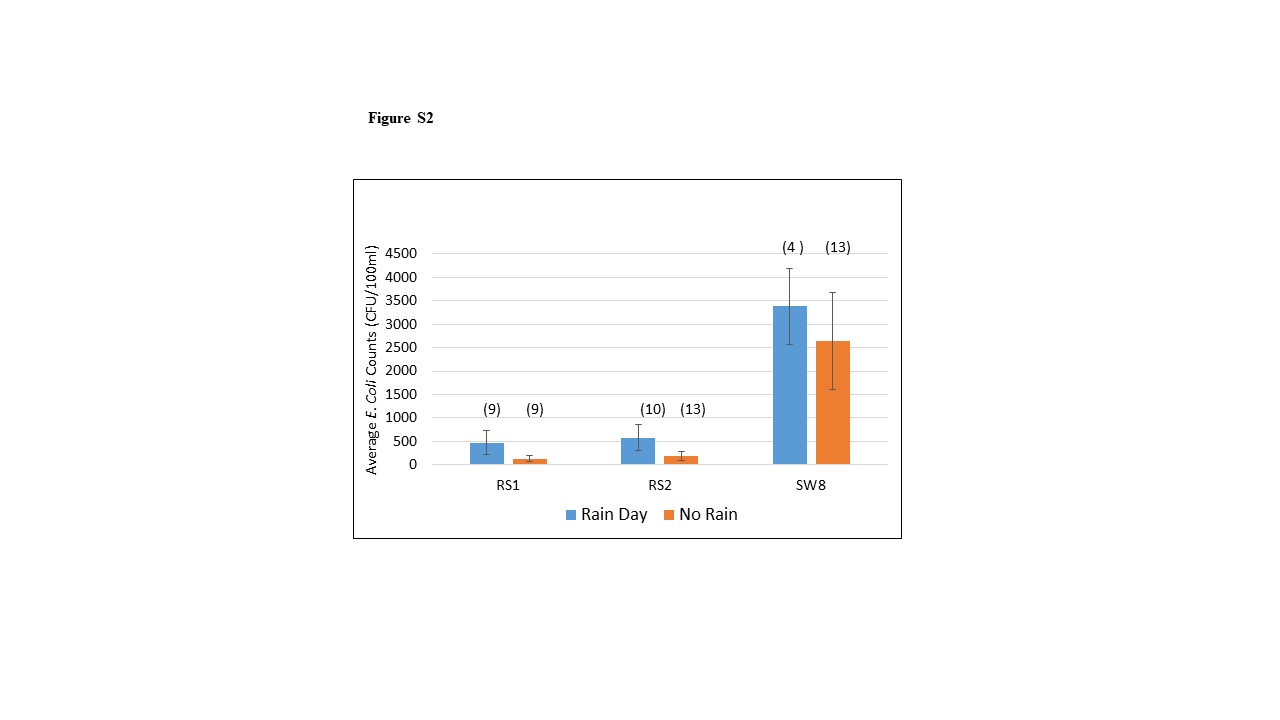

Supplement: FIGURE S2 — Impact of Rain events on total counts of E. coli. Total number of E. coli in CFUs was determined at three of the sites, RS1, RS2, and SW8 before and after a rainfall event. The number of times (N) this was determined at each site is indicated in the figure. [file Image_3.JPEG]
